# Process evaluation in complex public health intervention studies: the need for guidance

**DOI:** 10.1136/jech-2013-202869

**Published:** 2013-09-10

**Authors:** Graham Moore, Suzanne Audrey, Mary Barker, Lyndal Bond, Chris Bonell, Cyrus Cooper, Wendy Hardeman, Laurence Moore, Alicia O'Cathain, Tannaze Tinati, Danny Wight, Janis Baird

**Affiliations:** 1 DECIPHer UKCRC Public Health Research Centre of Excellence, School of Social Sciences, Cardiff University, Cardiff, UK; 2 DECIPHer UKCRC Public Health Research Centre of Excellence, School of Social and Community Medicine, University of Bristol, Bristol, UK; 3 MRC Lifecourse Epidemiology Unit, University of Southampton, Southampton, UK; 4 Centre of Excellence in Intervention and Prevention Science, Melbourne, VIC Australia; 5 Social Science Research Unit, Institute of Education, University of London, London, UK; 6 The Primary Care Unit, Department of Public Health and Primary Care, University of Cambridge, Cambridge, UK; 7 Cardiff University Schools of Social Sciences and Medicine, Cardiff, UK; 8 DECIPHer UKCRC Public Health Research Centre of Excellence, Cardiff, UK; 9 School of Health and Related Research, University of Sheffield, Sheffield, UK; 10 MRC Social and Public Health Sciences Unit, GLASGOW, UK

**Keywords:** PUBLIC HEALTH, RESEARCH METHODS, METHODOLOGY

## Why is process evaluation needed in complex public health intervention research?

Public health interventions aim to improve the health of populations or at-risk subgroups. Problems targeted by such interventions, such as diet and smoking, involve complex multifactorial aetiology. Interventions will often aim to address more than one cause simultaneously, targeting factors at multiple levels (eg, individual, interpersonal, organisational), and comprising several components which interact to affect more than one outcome.^1^ They will often be delivered in systems which respond in unpredictable ways to the new intervention.^2^ Recognition is growing that evaluations need to understand this complexity if they are to inform future intervention development, or efforts to apply the same intervention in another setting or population.^1^ Achieving this will require evaluators to move beyond a ‘does it work?’ focus, towards combining outcomes and process evaluation. There is no such thing as a typical process evaluation, with the term applied to studies which range from a few simple quantitative items on satisfaction, to complex mixed-method studies exploring issues such as the process of implementation, or contextual influences on implementation and outcomes. As recognised within MRC guidance for evaluating complex interventions, process evaluation may be used to ‘assess fidelity and quality of *implementation*, clarify *causal mechanisms* and identify *contextual factors* associated with variation in outcomes’.^1^ This paper briefly discusses each of these core aims for process evaluation, before describing current Medical Research Council (MRC) Population Health Sciences Research Network (PHSRN) funded work to develop guidance for process evaluations of complex public health interventions.

### Intervention implementation

An important role for process evaluations is to examine the quantity and quality of what was actually implemented in practice, and why. This may inform implementation of similar interventions elsewhere, and facilitate interpretation of intervention outcomes. While notions of standardisation are central to implementation assessment, the nature of standardisation required in complex interventions is debated. Hawe and colleagues argue that tailoring intervention from across contexts may ensure greater consistency with intended functions.^3^ Others argue that when we know little about which are core active ingredients, we might be tempted to make adaptations which inhibit effectiveness.^4^ Durlak and DuPre argue that ‘core components should receive emphasis in terms of fidelity’, with remaining components allowed to vary.^5^ All these positions highlight the need for implementation assessments within process evaluations to be guided by a thorough knowledge of the intervention theory, in terms of which components are expected to do what, and how changes during implementation may impede or optimise functioning. While assessments of feasibility will likely be made in exploratory evaluation phases, new challenges in implementation may need to be understood as an intervention moves into a wider range of contexts for a larger scale evaluation of effectiveness. Exploring issues such as acceptability may also enable evaluators to better understand intervention delivery and reach.

### Causal mechanisms

All public health interventions reflect implicit or explicit theories regarding how a course of action will solve a perceived problem. However, the contribution of outcomes evaluations to theory development is often limited by failures to clearly articulate the assumptions being made, as well as naive tendencies to presume that what was implemented was consistent with these assumptions, as discussed above. MRC guidance argues that only through close scrutiny of causal mechanisms is it possible for evaluation to contribute to developing more effective interventions, and provide insights into how findings might be transferred across settings and populations.^1^ Some early frameworks for process evaluations in public health focused predominantly on implementation and context, paying more limited attention to causal mechanisms.^6^ However, the need for evaluations to test and develop intervention theory, rather than simply provide pass or fail assessments of effectiveness, is recognised within approaches such as Realistic Evaluation,^7^ the more recently proposed concept of Realist Trials,^8^ and recent recommendations for process evaluation within cluster randomised trials.^9^ Process evaluations may usefully combine quantitative assessments of hypothesised mediators with qualitative investigation of unanticipated or complex causal pathways, and unanticipated consequences.

### Contextual factors

Understanding the contexts in which complex interventions are delivered and received is critical to explaining why they do or do not work, or how we might expect impacts to differ if implemented elsewhere. ‘Context’ may include any factors which are external to the intervention, but which may impede or strengthen the effects of an intervention. This may include factors which existed prior to the intervention, or factors which emerge during its implementation. While early process evaluation frameworks emphasised roles of context in shaping implementation,^6^ contextual factors may also moderate outcomes. Implementation will often require complex behavioural changes among implementers, and may be influenced by factors such as their circumstances, skills and attitudes.^7^ ^10^ ^11^ Similarly, participants do not receive interventions passively but interact with them in ways influenced by their circumstances, attitudes, beliefs, social norms and resources. The causal pathways of problems targeted by public health intervention will differ from one time and place to another. Hence, the same intervention may have different consequences if implemented in a different setting, or among different subgroups. Even where an intervention itself is relatively simple, its causal processes and relationship with its context may still be considered complex.

## Why is guidance needed?

In 2008 the MRC updated their guidance on the development and evaluation of complex interventions. As described above, in a change in emphasis from the earlier version published in 2000, revised guidance called for definitive evaluation to combine evaluation of outcomes with that of process.^1^ It did not, however, offer detail on *how* to conduct process evaluation. Existing frameworks offer some assistance to researchers conducting process evaluations.^6^ However, these tend to focus on identifying priority aims for process evaluations, rather than providing detailed guidance on how to plan, design or conduct a process evaluation.

The shortage of guidance to assist researchers, grant referees or journal reviewers in the design, conduct or assessment of process evaluation means that process evaluation may be planned in an ad hoc fashion. Hence, while process evaluation is an increasingly common component of grant applications, study protocols and published evaluations of public health interventions approaches are highly variable.^9^ The appearance of variability in approaches is perhaps heightened further by inconsistency in reporting, perhaps stemming from challenges reporting process data in a single article while remaining within journal word limits. Furthermore, there is often limited integration between process and outcomes studies.^12^

## Developing guidance for process evaluations of complex public health interventions

In November 2010 an MRC PHSRN-funded workshop discussed the need for guidance on process evaluation in complex public health intervention studies. The workshop was conceived by a number of groups undertaking complex intervention research that had identified the need to draw on collective expertise in developing process evaluation. Workshop participants, who were predominantly public health researchers and policy makers, were strongly in favour of the development of guidance to assist them in their research. There was also consensus that funders and reviewers of grant applications would benefit from guidance to assist peer review.

Following the workshop, the MRC PHSRN is supporting development of guidance for process evaluation of complex public health interventions, focusing predominantly upon process evaluations within trials or other outcomes evaluations. The authors form the group leading the development of guidance. It will focus upon priorities for process evaluation identified within the MRC framework for complex interventions, in terms of understanding implementation, causal mechanisms and the relationship of interventions with their contexts. The guidance is being developed through an iterative process of literature review, reflection on detailed case studies of process evaluations in which the guideline development group has been involved, and consultation with stakeholders including researchers, journal editors, and policy and practice partners. The aim of this endeavour is to provide public health researchers, policy makers, research funders and journal editors with guidance in designing, conducting and appraising process evaluations of complex public health interventions.
